# Reply to Maffulli, N.; Spiezia, F. Comment on “Foti et al. Identification of Achille’s Tendon Tears: Diagnostic Accuracy of Dual-Energy CT with Respect to MRI. *J. Clin. Med.* 2024, *13*, 4426”

**DOI:** 10.3390/jcm13237323

**Published:** 2024-12-02

**Authors:** Giovanni Foti, Luca Bortoli, Matteo Tronu, Sabrina Montefusco, Gerardo Serra, Roberto Filippini, Venanzio Iacono

**Affiliations:** 1 Department of Radiology, IRCCS Sacro Cuore Hospital, 37042 Negrar, Italy; 2 Department of Radiology, Verona University Hospital, 37126 Verona, Italy; lbortoli95@gmail.com (L.B.); matteo.tronu.94@gmail.com (M.T.); sab.montefusco@gmail.com (S.M.); 3 Department of Anesthesia and Analgesic Therapy, IRCCS Sacro Cuore Don Calabria Hospital, 37024 Negrar, Italy; gerardo.serra@sacrocuore.it; 4 Department of Sports Medicine, IRCCS Sacro Cuore Hospital, 37042 Negrar, Italy; roberto.filippini@sacrocuore.it; 5 Department of Orthopaedics, IRCCS Ospedale Sacro Cuore Don Calabria, 37024 Negrar, Italy; venanzio.iacono@sacrocuore.it

I need to thank my colleagues for their valuable comments on the recently published paper entitled “Identification of Achille’s Tendon Tears: Diagnostic Accuracy of Dual-Energy CT with Respect to MRI”. The clinical topic of the study concerns the evaluation of the Achilles tendon, a structure characterized by complex biomechanics. Its pathology, which is multifactorial and has various facets, can be challenging to assess in daily practice [1,2].

I fully agree with the analysis conducted and recognize the importance of clinical examination, ultrasound, and MRI for the pre- and post-surgical assessment [3,4,5,6,7,8].

Indeed, in my daily practice as a radiologist, I always rely on ultrasound and MRI for the management of patients with Achilles tendon pathology. These tests surely represent the reference standard, in my opinion. 

However, the purpose of this feasibility study was to evaluate the possibility of using DECT for the assessment of tendon tears. Although DECT has been proposed for imaging the anterior cruciate ligament [8,9,10], the assessment of Achille’s tendon represents a new topic for DECT, especially because it was carried out with the use of a non-specific application. 

Only through small steps, including analyses like ours, will we be able to understand whether DECT (and the future machines that will follow) can truly play a role in the evaluation of tendon and ligament pathologies.

Conversely, I must express my disagreement regarding the limitations mentioned: nowadays, DECT scanners are largely available in Western countries. These scanners are cheaper than MRI ones, with inferior reimbursements (less impact on the healthcare system), and the scanners are much faster, with an MSK acquisition lasting only a few seconds.

Also, radiation exposure issues are not significant, except in very young patients, because the latest generation scanners, and even more so the photon counting scanners that have been on the market for about 2 years (representing the most recent evolution of DECT), allow for very low doses that are negligible in adult patients [11]. Also, radiation burden is usually neglectable in the foot. 

On the contrary, CT offers additional advantages: in comparison to MRI, it is faster, less expensive, and allows for optimal evaluation of bone trabeculation and small intra- or peri-articular or tendinous calcifications, with spatial resolution reaching up to 0.2 mm. There are no issues with claustrophobia or pacemakers. Moreover, DECT is the best method for reducing artifacts caused by synthetic metal components or prostheses [12,13]. With respect to the US, DECT is once again capable of evaluating the bone with great detail, encompassing the possibility of detecting bone marrow edema (BME) and bone marrow lesions around the joints [14,15,16]. Also, DECT arthrography can take advantage of the multifactorial approach based on several specific applications [17]. For these reasons, DECT has been successfully employed for diagnosing several entities in the lower limb, including traumatic and non-traumatic BME, such as in transient bone marrow edema syndromes, in stress or insufficiency fractures, or even in infective diseases such as osteomyelitis [18,19,20]. In these settings, it is not a mistake, in my opinion, to invest in DECT technology to potentially achieve, in the not-too-distant future, a rapid and readily available method to be offered to patients who, for various reasons, do not have access to MRI or cannot be managed with ultrasound alone.

## References

[1] Foti G., Bortoli L., Tronu M., Montefusco S., Serra G., Filippini R., Iacono V. (2024). Identification of Achille’s Tendon Tears: Diagnostic Accuracy of Dual-Energy CT with Respect to MRI. J. Clin. Med..

[2] Maffulli N., Spiezia F. (2024). Comment on Foti et al. Identification of Achille’s Tendon Tears: Diagnostic Accuracy of Dual-Energy CT with Respect to MRI. *J. Clin. Med.* 2024, *13*, 4426. J. Clin. Med..

[3] Reiman M., Burgi C., Strube E., Prue K., Ray K., Elliott A., Goode A. (2014). The utility of clinical measures for the diagnosis of achilles 50 tendon injuries: A systematic review with meta-analysis. J. Athl. Train..

[4] Thompson T.C. (1962). A Test for Rupture of the Tendo Achillis. Acta Orthop. Scand..

[5] Copeland S.A. (1990). Rupture of the Achilles tendon: A new clinical test. Ann. R. Coll. Surg. Engl..

[6] Chianca V., Zappia M., Oliva F., Luca B., Maffulli N. (2020). Post-operative MRI and US appearance of the Achilles tendons. J. Ultrasound.

[7] Keene J.S., Lash E.G., Fisher D.R., De Smet A.A. (1989). Magnetic resonance imaging of Achilles tendon ruptures. Am. J. Sports Med..

[8] Maffulli N., Dymond N.P., Capasso G. (1989). Ultrasonographic findings in subcutaneous rupture of Achilles tendon. J. Sports Med. Phys. Fit..

[9] Liu D., Hu P., Cai Z.J., Lu W.H., Pan L.Y., Liu X., Peng X.J., Li Y.S., Xiao W.F. (2023). Valid and reliable diagnostic performance of dual-energy CT in anterior cruciate ligament rupture. Eur. Radiol..

[10] Gruenewald L.D., Koch V., Martin S.S., Yel I., Mahmoudi S., Bernatz S., Eichler K., Alizadeh L.S., D’Angelo T., Mazziotti S. (2023). Diagnostic value of DECT-based colored collagen maps for the assessment of cruciate ligaments in patients with acute trauma. Eur. Radiol..

[11] Liu L.P., Shapira N., Chen A.A., Shinohara R.T., Sahbaee P., Schnall M., Litt H.I., Noël P.B. (2022). First-generation clinical dual-source photon-counting CT: Ultra-low-dose quantitative spectral imaging. Eur. Radiol..

[12] Foti G., Longo C., D’Onofrio M., Natali S., Piovan G., Oliboni E., Iacono V., Guerriero M., Zorzi C. (2023). Dual-Energy CT for Detecting Painful Knee Prosthesis Loosening. Radiology.

[13] Foti G., Fighera A., Campacci A., Natali S., Guerriero M., Zorzi C., Carbognin G. (2021). Diagnostic Performance of Dual-Energy CT for Detecting Painful Hip Prosthesis Loosening. Radiology.

[14] Foti G., Guerriero M., Faccioli N., Fighera A., Romano L., Zorzi C., Carbognin G. (2021). Identification of bone marrow edema around the ankle joint in non-traumatic patients: Diagnostic accuracy of dual-energy computed tomography. Clin. Imaging.

[15] Foti G., Sanfilippo L., Longo C., Oliboni E., De Santis N., Iacono V., Serra G., Guerriero M., Filippini R. (2024). Diagnostic Accuracy of Dual-Energy CT for Bone Stress Injury of the Lower Limb. Radiology.

[16] Guggenberger R., Gnannt R., Hodler J., Krauss B., Wanner G.A., Csuka E., Payne B., Frauenfelder T., Andreisek G., Alkadhi H. (2012). Diagnostic performance of dual-energy CT for the detection of traumatic bone marrow lesions in the ankle: Comparison with MR imaging. Radiology.

[17] Foti G., Booz C., Buculo G.M., Oliboni E., Longo C., Avanzi P., Campacci A., Zorzi CDual-Energy C.T. (2023). Arthrography: Advanced Muscolo-Skelatal Applications in Clinical Practice. Tomography.

[18] Foti G., Serra G., Iacono V., Zorzi C. (2021). Identification of Traumatic Bone Marrow Oedema: The Pearls and Pitfalls of Dual-Energy CT (DECT). Tomography.

[19] Foti G., Serra G., Iacono V., Marocco S., Bertoli G., Gori S., Zorzi C. (2021). Identification of Non-Traumatic Bone Marrow Oedema: The Pearls and Pitfalls of Dual-Energy CT (DECT). Tomography.

[20] Foti G., Longo C., Sorgato C., Oliboni E.S., Mazzi C., Motta L., Bertoli G., Marocco S. (2023). Osteomyelitis of the Lower Limb: Diagnostic Accuracy of Dual-Energy CT versus MRI. Diagnostics.

